# Impact of Microenvironmental Changes during Degeneration on Intervertebral Disc Progenitor Cells: A Comparison with Mesenchymal Stem Cells

**DOI:** 10.3390/bioengineering9040148

**Published:** 2022-04-01

**Authors:** Feng-Juan Lyu

**Affiliations:** Joint Center for Regenerative Medicine Research of South China University of Technology-the University of Western Australia, School of Medicine, South China University of Technology, Guangzhou 510006, China; lufj0@scut.edu.cn

**Keywords:** intervertebral disc, degeneration, disc progenitor cells, mesenchymal stem cells, microenvironment

## Abstract

Intervertebral disc (IVD) degeneration occurs with natural ageing and is linked to low back pain, a common disease. As an avascular tissue, the microenvironment inside the IVD is harsh. During degeneration, the condition becomes even more compromised, presenting a significant challenge to the survival and function of the resident cells, as well as to any regeneration attempts using cell implantation. Mesenchymal stem cells (MSCs) have been proposed as a candidate stem cell tool for IVD regeneration. Recently, endogenous IVD progenitor cells have been identified inside the IVD, highlighting their potential for self-repair. IVD progenitor cells have properties similar to MSCs, with minor differences in potency and surface marker expression. Currently, it is unclear how IVD progenitor cells react to microenvironmental factors and in what ways they possibly behave differently to MSCs. Here, we first summarized the microenvironmental factors presented in the IVD and their changes during degeneration. Then, we analyzed the available studies on the responses of IVD progenitor cells and MSCs to these factors, and made comparisons between these two types of cells, when possible, in an attempt to achieve a clear understanding of the characteristics of IVD progenitor cells when compared to MSCs; as well as, to provide possible clues to cell fate after implantation, which may facilitate future manipulation and design of IVD regeneration studies.

## 1. Introduction

Intervertebral discs (IVD) are semi-hard joint tissues in the spine, with a major contribution to spine motion. The nucleus pulposus (NP) occupies the center of the IVD and is encased by an outer ring of annulus fibrosus (AF) and two capping cartilaginous endplates (CEP). IVD degeneration contributes to low back pain, which is a major cause of disability [1]. Moreover, degenerated IVD can cause compression of the spinal cord and nerve root, and lead to myelopathy and sciatica.

Currently, conservative therapies are recommended to control mild to moderate discogenic low back pain. Conservative therapies include non-drug treatments and drug therapy. Non-drug treatments include physical therapy, acupuncture, massage, exercise such as yoga and tai chi, and spinal traction. The available drugs include acetaminophen, nonsteroidal anti-inflammatory drugs (NSAIDs), skeletal muscle relaxants, corticosteroids, tricyclic antidepressants, anti-epileptic drugs, tramadol, and temporal administration of opioid drugs for the management of medium to severe pain [2,3]. For patients with persistent and unbearable pain, and who have failed conservative therapies, surgical procedures are considered, which include nucleus pulposus enucleation, nucleolysis, nucleoplasty, percutaneous laser disc decompression (PLDD), artificial disc replacement, and spinal fusion, Surgical therapies have shown some benefits (i.e., pain relief), with most patients showing evidence of short-term pain improvement, but many lack evidence of sustained benefit and complete remission. All these surgeries aim to achieve pain relief by tissue elimination or tissue fixation, instead of recovering the biological structure and function of the degenerated IVD. This process changes the biomechanics of the native spine, may contribute to further disc disease, and cannot reverse the negative cascade in the IVD [4].

Recently, regeneration medicine has captured the focus of research interest, to investigate the possibility of applying stem cells for IVD repair. As the precursor of mesenchymal lineages, mesenchymal stem cells (MSCs) are the main type of stem cell tool. In addition, endogenous progenitor cells have been recently identified in the IVD, including in the NP, CEP, and AF, highlighting the emergence of a novel type of progenitor cell for IVD repair [5]. These cells share some characteristics with MSCs and are often referred to as MSC-like cells. However, it is well known that the local microenvironment has a great impact on stem cell differentiation and function [6]. The harsh microenvironment inside the disc, which can become more serious during degeneration or ageing, can have a strong impact on the behavior and function of implanted cells; thus, presenting a rigorous challenge to the engraftment or differentiation of stem or progenitor cells. It remains unclear which factors outweigh the others in determining the fate of implanted cells, and should receive more attention from researchers.

In this review, we first briefly summarize the current stem cell sources for IVD repair. Then, we detail the factors which define the IVD microenvironment, and their changes during degeneration. We, subsequently, put our focus on the possible impact of the deteriorated microenvironment on disc progenitor cells, with an emphasis on NP progenitor cells. Since these studies are limited due to the novelty of this research field, we also analyze the influence of a degenerated disc microenvironment on MSCs, especially bone marrow-derived MSCs (BM-MSCs), as a reference and to compare with the impacts on disc progenitor cells; to clarify the similarities and differences between disc progenitor cells as compared to MSCs, with respect to their responses to common disc microenvironment factors, and to provide some clues for the strategic manipulation of disc progenitor cells or MSCs for IVD regeneration.

## 2. The IVD Microenvironment

The microenvironment in the disc is complicated. Due to its avascular nature, the nearest blood vessel can be as far as 8 mm [7]. Therefore, nutrients transported by capillaries in the adjacent vertebral bodies can only inefficiently permeate into the NP through the upper and lower CEP [8], and it is also difficult to transport metabolic waste out of the disc. In consequence, these factors constitute a straitened circumstance for the cells inside.

The consumption of glucose during anaerobic glycolysis results in a steep nutrient gradient towards the core of the IVD [8]. As a result, the center of the NP is characterized by low glucose and a high content of lactic acid, due to glucose consumption. The glucose level is reported to be around 1 mmol/L [9], and lactate concentrations are reported to be around 5 mmol/L [9,10]. The pH level in a healthy disc ranges between 7.2 and 7.0 [11] and is around 0.5 pH units below that of the surrounding fluids [12]. The physiological oxygen tension in human NP is low, ranging from 5 to 150 mm·Hg [10], with the average concentration measuring around 2%; lower than the levels in many other organs and tissues. The extracellular matrix (ECM) in the NP is mainly composed of collagens and proteoglycans. In the NP, the ECM is rich in collagen II and aggrecan. The collagens build the structure and tensile strength, while proteoglycans provide viscoelasticity, absorb water, and provide resistance to compression. Proteoglycans comprise 50% of the NP dry weight in children, and deliver an osmotic pressure that varies from ~430 mOsm/L to ~496 mOsm/L [13] in the IVD; while, aggrecan is the major type [14].

**Figure 1:**
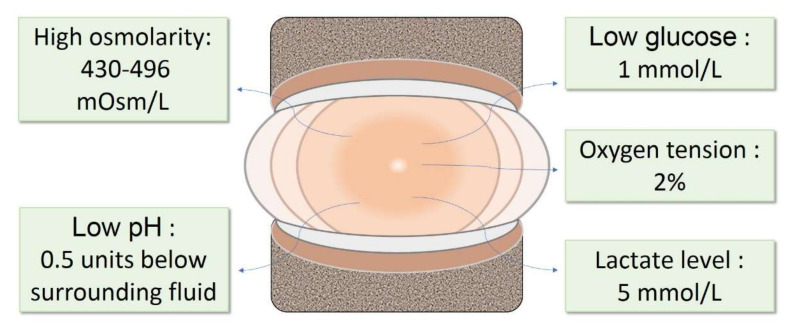
The physiochemical microenvironment in healthy NP. The NP in healthy IVDs is defined by low glucose, low pH, low oxygen tension, high lactate level, and high osmolarity. IVD: intervertebral disc; NP: nucleus pulposus.

In summary, the IVD niche is defined by low glucose, low pH, low oxygen tension, high osmolarity, and a tensile and moist ECM (Figure 1).

## 3. Changes in IVD Microenvironment during Degeneration

During IVD degeneration, changes occur in all the components of the IVD. The CEP thins and calcifies during IVD degeneration, making the diffusion routes of nutrient supply become sparser in aged NP. In consequence, the inner microenvironment of the IVD becomes more inhospitable and catabolic.

First, calcified CEP and disc deformation lead to a decreased glucose concentration in the IVD [15]. When the permeability of the CEP dropped by 50%, the glucose level was only half of that in healthy NP [9]. Matrix acidity drops drastically during disc degeneration as a result of proton accumulation, with pH levels of 6.5–5.7 usually recorded [16,17]. The oxygen tension drops from 2% in healthy NP, to ~1% in degenerated NP [10]. In addition, degenerated IVDs are demonstrated to be in a chronic inflammatory state [18], with increased expression of multiple pro-inflammatory cytokines. These include interleukin (IL) 1, matrix metalloproteinase (MMP) 10 [19], MMP12 [20], cyclooxygenase 2 (COX-2) [21], IL-8, tumor necrosis factor-α (TNF-α) [22], IL-10 [23], IL-2, IL-4, and IL-17, etc. [24,25], which may be strongly connected to pain production [26]. Of these, IL-1 is of particular interest, since IL-1 plays a more important role in mediating disc matrix degradation than TNF-α [27,28]. IL-1β also leads to production of MMPs and pain mediators, such as the eicosanoid prostaglandin E2, by human IVD cells [29]. Besides, disc degeneration leads to a stiffer ECM. The shear stiffness of human NP, measured by magnetic resonance elastography, increased from 12.5 kPa at Pfirrmann degeneration grade1 to 16.5 kPa at Pfirrmann degeneration grade 5 [30]. Similarly, collagen fibers became stiffer and thicker in rabbit NP after needle puncture was introduced [31]. The production of aggrecan is decreased [32], with increased fragmentation [33,34] and other ECM proteins [35]. As a result, the loss of aggrecan in degenerated IVD causes decreased osmolarity, to around 300 mOsm/L [36].

In summary, the disc degeneration caused by injury or ageing results in stiffer ECM, reduced glucose and osmolarity, increased acidity, hypoxia, and inflammation.

## 4. Stem Cells Exploited for IVD Repair

**Figure 2:**
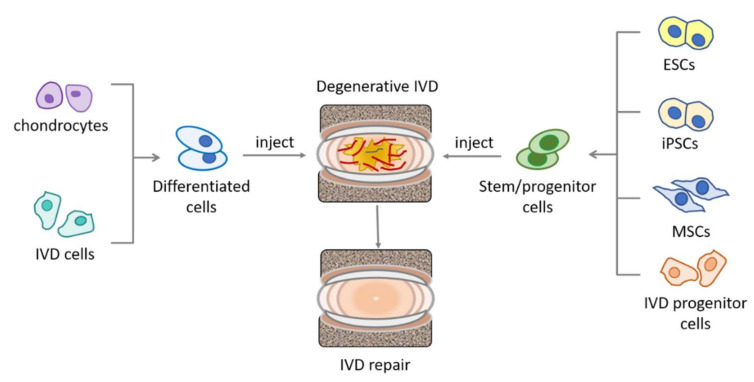
Sources of different cell types for IVD repair. Differentiated cells, including chondrocytes and IVD cells, and stem/progenitor cells, including ESCs, MSCs, iPSCs, and the recently identified IVD progenitor cells, have been explored for IVD repair. MSCs: mesenchymal stem cells; ESCs: embryonic stem cells; iPSCs: induced pluripotent stem cells.

Cells, including differentiated cells, as well as various types of stem cells, have been investigated for their potential in IVD repair studies (Figure 2). Differentiated cells include chondrocytes [37] and IVD cells [38,39]. Stem cells, due to their high self-renewal capacity and differentiation potential, stand in the spot light of modern regenerative medicine, as they provide the possibility of replenishing the diminishing cell population in the host tissue. To date, the type of stem cells exploited for IVD repair have included mesenchymal stem cells (MSCs), embryonic stem cells [40], and induced pluripotent stem cells [41], with MSCs being the most intensively investigated type. MSCs are the precursors of mesenchymal lineages, defined by their tri-potency for generating osteoblasts, chondrocytes, and adipocytes. MSCs can be isolated from many tissues and organs [42,43] and some are postulated to originate from pericytes, with variations in their differentiation potency and cytokine secretion or gene expression profile associated with their host tissue microenvironment [44]. For example, bone marrow-derived MSCs have a series of upregulated genes related to antimicrobial activity and osteogenesis, when compared to umbilical cord derived MSCs, while the latter have more transcripts associated with matrix remodeling and angiogenesis [45]. Due to their high self-renewal capacity and multipotency, MSCs are being researched for regeneration of many types of tissue and organs, such as bone, cartilage [46], tendon [47], and IVD [31].

Resident tissue-specific progenitor cells are found in many types of tissues and organs, such as in the skin [48] and liver [49]. They play an important role in the maintenance of tissue homeostasis, by slowly repopulating the tissue under physiological conditions and repairing damage under pathological conditions. Recently, endogenous progenitor cells were also found in the IVD [5], including in the NP, AF, and CEP. They express typical markers of MSCs, including CD29, CD44, CD73, CD90, and CD105, and show differentiation potential to undergo adipogenesis, chondrogenesis, and osteogenesis to a similar level when compared to MSCs. Some of the progenitor cells were even found to express pluripotency markers, such as Sox2 [50], Oct3/4, and Nanog [51], at both gene and protein level. These findings regarding disc progenitor cells highlight the self-repair potential of the IVD, and suggest a new progenitor cell source suitable for IVD repair.

## 5. The Impact of the Disc Microenvironment on MSCs and Disc Progenitor Cells

Degeneration makes the already harsh IVD microenvironment more hostile for cell survival. It breaks the catabolic–anabolic balance and constitutes a challenge for any regeneration attempts. Understanding how the degenerated disc microenvironment may affect stem cells will provide a profound aid in designing repair strategies with stem or progenitor cells. Currently, disc progenitor cells have been referred to in many studies as MSC-like cells, since they possess similar characteristics to MSCs. However, the behavior of MSCs and disc progenitor cells in a degenerative disc microenvironment has not been studied or summarized in full, nor is it clear whether disc progenitor cells behave similarly, or have a unique response that differs from that of MSCs. Here we look into recent progress made in the activities of disc progenitors under a degenerative disc microenvironment, as well as those of MSCs, as a reference, with an attempt to provide guidance for future research and clinical applications of stem cells in degenerated IVD.

## 6. Low Glucose and Serum Deficiency

### 6.1. MSCs

Glucose is an essential nutrient, providing energy for the daily metabolic activities of cells. In general, the harm of low glucose is not severe for MSCs. The culturing of rat BM-MSCs under a IVD-like glucose concentration of 1 mg/mL had a higher ECM production of aggrecan and collagen 1 compared to high glucose culturing at 4.5 mg/mL [52]. Interestingly, 1 mg/mL low glucose did not reduce, but slightly promoted, MSC proliferation [52]. In another study, human BM-MSCs showed no changes in proliferation or growth factor secretion when treated with various concentrations of glucose, from 5.5 mmol to 30 mmol, for up to 48 h [53]. IVD-like glucose at 1 mg/mL slightly reduced the cell viability of human adipose-derived MSCs (AT-MSCs) to 73%, but enhanced the production of aggrecan to 170%, with 4.5 mg/mL glucose after 2 weeks of treatment [54]. Consistently, low glucose at 0.25 mg/mL resulted in the lowest apoptosis rate and the highest colony forming units (CFU) of rat BM-MSCs with varied glucose treatments, from 0.25 mg/mL to 4.5 mg/mL [55]. However, the highest glucose level, at 4.5 mg/mL, maintained the highest cell viability of MSCs [55]. Together, these studies suggest that IVD-like low glucose does no harm, if not any good, to the survival and proliferation of MSCs. However, BM-MSCs did not performed well under serum deprivation condition and showed signs of apoptosis [56,57], which indicates that in severely degenerated IVD, the survival of MSCs remains a challenging issue.

### 6.2. IVD Progenitor Cells

For IVD progenitor cells, the nutrient deficiency caused by the removal of serum in culture was shown to promote apoptosis in CEP progenitor cells [58]. However, another 2016 study [59] showed that at initial time points, deprivation of glucose and serum caused some of the NP cells to exhibit a transient semi-adherent morphology; however, these cells were alive and would have recovered with minimal evidence of cell death after the addition of serum. Interestingly, when compared to serum-free and glucose-free culture, while the inclusion of moderate glucose resulted in significantly greater numbers of live human NP cells, the addition of fetal bovine serum (FBS) did not enhance, and in fact reduced, the number of live cells [59]. MSCs, by contrast, had more live cells when FBS was added to a serum-free and glucose-free culture [59].

Taken together, these studies suggest that low glucose may not be a critical limiting parameter for either MSCs or disc progenitor cells. Serum deprivation may enhance apoptosis in BM-MSCs and CEP progenitor cells, but not in NP progenitor cells, suggesting that NP progenitor cells have a lower demand on serum when compared to others. Nevertheless, it should be noticed that these were all in vitro studies and assessed within a relatively short period of time. Whether this conclusion can be applied in vivo awaits further investigation. From the in vitro experimental evidence, low glucose is not a limiting factor for disc progenitor cells or MSCs.

## 7. Acidity

In the NP, the pH level drops from 7.2–7.0 [11] in healthy tissue, to 6.5–5.7 in degenerated tissue. Among all the adverse parameters within the IVD, an acid condition can impose a profound influence on the survival and function of internal or transplanted cells.

### 7.1. MSCs

For MSCs, in a 2008 study, an acidic pH at 6.8 led to decreased proliferation and viability, and reduced expression of aggrecan and collagen I [52] in rat BM-MSCs, when compared to a standard culture pH at 7.6. Furthermore, the same group also demonstrated that the degree of such an effect was positively associated with the level of acidity, from pH 7.4 to pH 6.5 [60]. The cell number of BM-MSCs decreased by 40% and 80%, while the cell viability decreased by 70% and 90%, respectively, at pH 6.8 and pH 6.5, when compared to that at pH 7.4, after 5 days of treatment [60]. For AT-MSCs, the drop of pH from 7.4 to 6.5 drastically inhibited the ECM expression (collagen I, collagen II, aggrecan [61]), upregulated catabolic enzymes, and reduced cell viability and proliferation [62]. In addition, AT-MSCs from young donors were found to respond similarly to acidity, but to a lesser extent, compared to those from older donors [61]; indicating that MSCs from young individuals are less sensitive to acidity. However, despite the negative effects, acidic treatment for a short period of time may stimulate the stemness properties of MSCs. A 2017 study found that a short treatment of low pH at 6.5–6.8 for 1–7 days made BM-MSCs stay in the G0 stage, expressing more stemness markers, and promoted the formation of spheroidal colony forming units (CFU-S) [63]. Currently, how acidity affects the differentiation potential of MSCs is not clear. Gao et al. reported that cariporide treatment, which can artificially create decreased intracellular pH, was able to promote the osteogenic differentiation of human UC-MSCs, while the adipogenesis potency was not affected [64]. However, Massa et al. reported that continuous exposure to acidic pH at 6.5–6.8 for 21 days impaired the osteogenic differentiation of BM-MSCs [63]. Thus, it seems that acidity can decrease cell viability and proliferation, and inhibit ECM production; whereas, a short treatment of acidity may stimulate the stemness of MSCs.

### 7.2. IVD Progenitor Cells

Similarly, it was reported that acidity is linked with impaired function of disc progenitor cells. In a 2014 study, acidic conditions at pH 6.8 to 6.5 decreased cell viability and proliferation; reduced the expression of ECM, including collagen I, collagen II, aggrecan, and tissue inhibitor of metalloproteinase (TIMP) -3; and upregulated the expression of catabolic enzymes, including MMP-2 and a disintegrin and metal loproteinase with thrombospondin motifs-4 (ADAMTS4), in NP progenitor cells isolated from Sprague-Dawley rats, when compared to standard culture conditions at pH 7.4 [62]. Likewise, in a 2017 study, a drop in pH from 7.4 to 6.2 increasingly inhibited cell proliferation and promoted the cell apoptosis of human NP progenitor cells [65]. The low pH also inhibited the expression of stemness markers, such as Notch1, Jagged, Nanog, and Oct4, and reduced the ECM synthesis of collagen I, collagen II, and aggrecan [65].

In conclusion, the impact of acidity is similar on both MSCs and disc progenitor cells. It decreases cell viability and proliferation, and inhibits ECM production. Although a short treatment of acidity may stimulate the stemness of MSCs, it is currently unclear whether this would have the same effect on disc progenitor cells.

## 8. Lactic Acid Accumulation

The acidic condition in the NP is due to the deposited lactic acid and insufficient clearing. Currently there are no reports on how IVD progenitor cells respond to lactic acid accumulation, so we have to draw on experience with MSCs. We found that the effect of lactate may depend on its concentration. In human BM-MSCs, a low lactate treatment at 5–10 mmol for 24 h led to anti-inflammatory and anti-apoptotic gene expression, including downregulation of IL-1β and IL-8, and upregulated cell growth and proliferation [66]. However, a high lactate concentration at 15 mmol showed the opposed effect of promoting inflammatory cytokine production in MSCs, such as IL-1β, IL-6, IL-8, and IL-26; and apoptotic related gene expression, such as Bcl-2 associated X protein, caspase 9 and nuclear factor κB (NFκB) [66]. Therefore, a low lactate concentration may stimulate an anti-inflammatory and anti-apoptotic profile, while high lactate concentrations may a stimulate inflammatory and apoptotic profile of MSCs. Considering that the concentration of lactic acid in healthy NP is around 5 mmol, which increases after degeneration, it can be postulated that high levels of lactate acid in degenerated discs may also be a leading cause of the induction of an inflammatory profile and apoptosis of disc progenitor cells.

## 9. Hypoxia

### 9.1. MSCs

MSCs seem to have some resistance to the apoptotic and proliferation inhibition effect of hypoxia. Huang et al. reported that monkey BM-MSCs did not exhibit signs of apoptosis after 10 days in 3.5% O_2_ [67]. Moreover, human AT-MSCs under 1.5 and 2% O_2_ displayed a lower rate of apoptotic events when compared to those under normoxia [68,69]. Short-term hypoxic treatment at 2–5% O_2_ for 48 h could enhance the proliferation of human and porcine BM-MSCs [70]. Although middle-term culture in 2% O_2_ slowed the proliferation of rat AT-MSCs [71] and human BM-MSCs [70,72], when assessed from day 7 to day 14, it was conversely found that the proliferation recovered and the total cell number was similar to those in normoxia after 30 days [72], and the cells retained differentiation capabilities for at least seven passages [73].

Hypoxia may stimulate the stemness phenotypes of MSCs, represented by CFU frequency and stemness marker expression. The frequency of fibroblastic colony-forming-units (CFU-F) formed by human BM-MSCs was increased, from 10% at day 1 to 14% at day 30, in 2% O_2_ [72]. The expression of stemness markers (Oct4, Rex1) was also higher under hypoxia than under normoxia [72]. This is in agreement with some later studies on human AT-MSCs, suggesting increased expression of stemness genes under hypoxia [74,75]. A further study revealed that hypoxic human BM-MSCs lost contact inhibition and formed multiple cell layers in culture [73]. This is not indicative of tumorigenicity, since the hypoxic cultured MSCs had normal karyotyping and intact genetic integrity, even after 100 population doublings [76]. Taken together, these findings indicate that hypoxia can preserve the stemness of MSCs.

Hypoxia also affects the differentiation potential of MSCs. Hypoxia stimulated the chondrogenic potential of rat BM-MSCs [77], porcine BM-MSCs [78], and rat AT-MSCs [71], mainly represented by the expression of aggrecan, collagen II, and Sox9. In the literature, the effect of hypoxia on the osteogenesis and adipogenesis of MSC is not consistent. Some studies reported that hypoxic cultured BM-MSCs expressed a higher level of osteogenesis and adipogenesis markers than the normoxic group after induction [72,79,80], while in other studies, hypoxic culture (2% and 5% O_2_) was found to impair the osteogenic potential of BM-MSCs [81,82] and AT-MSCs [74,83,84], represented by lower expression of osteogenic markers, such as osteocalcin, Runx2, or alkaline phosphatase. In adipogenic induction, some studies found that a 2–5% O_2_ culture condition made AT-MSCs express low levels of adipogenesis markers (such as PPAR-γ, LPL, and FABP4) compared to those under normoxia [74,85].

Hypoxia is found to promote the anti-inflammatory effect of MSCs. Hypoxia at 2–5% O_2_ for 48 h and 10 days suppressed the production of IL-8, and hypoxia at 2–5% O_2_ for 48 h elevated IL-1ra and granulocyte-macrophage colony stimulating factor (GM-CSF), the anti-inflammatory cytokines, in human and porcine BM-MSCs [70], suggesting that hypoxia can help MSCs to exert an anti-inflammatory effect.

### 9.2. IVD Progenitor Cells

Unlike MSCs, the apoptosis rate and proliferation of IVD progenitor cells are severely affected by hypoxia. IVD progenitor cells isolated from Rhesus monkey proliferated at a higher rate under 3.5% oxygen than those under a normoxic condition; however, this did not last long; they showed decelerated growth from the fifth day, and eventually underwent apoptosis by the tenth day in hypoxia [67]. Similarly, rat NP progenitor cells also showed decreased viability and proliferation after being cultured for 7–14 days in 2% O_2_ [71]_._ These findings indicate that hypoxia can temporarily stimulate the proliferation of disc progenitor cells, but that it induces apoptosis in the long turn. Similarly to MSCs, the differentiation potency of disc progenitor cells is affected by hypoxia. A 2013 study showed that hypoxia at 2% O_2_ promoted the chondrogenic differentiation of rat NP progenitor cells [71], indicating that hypoxia promotes chondrogenesis of disc progenitor cells. Both hypoxic treatment at 1% O_2_ [86] and the overexpression of hypoxia-inducible factor 1α [87] downregulated collagen I and Runx2 expression, and reduced the mineral deposition and alkaline phosphatase activity of human CEP progenitor cells, implying that hypoxia can impair the osteogenesis of disc progenitor cells. To summarize, hypoxia promotes the chondrogenic potential and inhibits the osteogenic potential of disc progenitor cells. Short-term hypoxia can stimulate proliferation, while long-term hypoxia leads to apoptosis of disc progenitor cells.

To sum up, hypoxia has a complex effect on progenitor/stem cells. It can stimulate chondrogenesis and promote an anti-inflammatory effect of MSCs. It promotes chondrogenesis and inhibits osteogenesis in disc progenitor cells. Hypoxia does not induce a apoptotic and proliferation inhibitory effect on MSCs, but does so for disc progenitor cells. An interesting finding is that hypoxia can stimulate the stemness phenotypes of MSCs. It is currently not clear whether a short treatment of hypoxia in disc progenitor cells may achieve the same effect.

## 10. Matrix Stiffness and Elasticity

Recent evidence suggests that the stiffness and elasticity of ECM mainly modulate the differentiation potential of stem/progenitor cells.

### 10.1. MSCs

Matrix stiffness mainly influences the differentiation efficiency of MSCs. Substrates with lower stiffness can elevate the expression of ACAN, Sox9, COL2, and proteoglycan during chondrogenic induction of MSCs, indicating an effect by promoting chondrogenesis [88,89]. In contrast, osteogenesis was elevated on stiffer substrates, represented by the expression of RUNX2, alkaline phosphatase specific activity, osteocalcin, and osteoprotegerin. [88,90,91]. The effect of matrix stiffness on the endothelial differentiation of MSCs is contradictory in the literature. One study reported that a stiffer matrix (15%) made from gelatin methacrylate hydrogel significantly reduced endothelial differentiation, represented by the expression of CD31, vascular endothelial growth factor (VEGF), and CD34 in human BM-MSCs, compared to soft matrices (7.5% and 10%) [91]. However, another study found that stiffer substrates, with an average surface modulus of 7.82 MPa, had a higher induction of endothelial differentiation in human MSCs than softer substrates, at 3.8 or 1.44 MPa [92].

### 10.2. IVD Progenitor Cells

For disc progenitor cells, ECM microstructure in the IVD affects ECM secretion and the differentiation direction of disc progenitor cells. In a study on a synthesized hydrogel matrix with various stiffnesses, a soft matrix with a low shear storage modulus enhanced the chondrogenesis and proliferation; while, a stiff matrix with a high modulus promoted the osteogenic differentiation of porcine NP progenitor cells [93]. Similarly, the fiber orientation affected the disc progenitor cells. A culture in an electrospun scaffold with aligned fibers could promote the expression of collagen I and aggrecan, but not collagen II, in rabbit AF progenitor cells, when compared to one with unaligned fibers [94]. In addition, the elasticity of a scaffold may modulate disc progenitor cells. Zhu et al. prepared an electrospun fibrous scaffold with four different elastic moduli, and found that different the elasticities had no influence on the cell proliferation of rabbit AF progenitor cells [95]. However, the increased elasticity of material stimulated the expression of collagen I and inhibited the expression of collagen II and aggrecan in these cells [95]. Thus, as for MSCs, a stiff matrix also inhibits chondrogenesis and promotes osteogenesis of disc progenitor cells.

## 11. Osmolarity

Currently, the impact of decreased osmolarity on disc progenitor cells has not been investigated, so clues can only be found when drawing lessons from its impact on MSCs.

### MSCs

A 2010 study showed that no significant change was observed in the cell viability of rat BM-MSCs after being exposed to varying osmolarities, from 250 mOsm to 370 mOsm, for 24 h [96]. It should be noted that 24 h is a rather short treatment time to assess cell behavior, and the cell response may have been different after a longer exposure. When compared to the osmolarity at 280 mOsm in standard culture conditions, IVD-like high osmolarity at 485 mOsm resulted in decreased proliferation and viability, and lower expression of aggrecan and collagen I [52] in rat BM-MSCs. For human AT-MSCs, IVD-like osmolarity at 485 mOsm significantly decreased cell viability to 50% and inhibited cell proliferation to 25%, as well as the production of collagen I to 60% and aggrecan to 7%, when compared to an osmolarity at 280 mOsm, after 2 weeks of treatment [54]. Again, the AT-MSCs from young donors behaved similarly to the cells from mature donors, but to a lesser extent.

These findings indicate that high osmolarity impairs the survival, proliferation, and function of MSCs. Considering that healthy IVD has a high osmolarity, the drop during degeneration is beneficial to MSCs, and might have the same effect on disc progenitor cells.

## 12. Inflammatory Factors

### 12.1. MSCs

MSCs are known for their effects in dampening inflammation in various types of tissue inflammation [97,98,99]. They can exert this function through recruiting monocytes to suppress inflammation [100], or by secreting extracellular vesicles [101], in which TNF-stimulated gene 6 (TSG-6) is an important mediator [102].

### 12.2. IVD Progenitor Cells

Disc progenitor cells seem to differ from MSCs, in that no evidence has shown that they have any anti-inflammatory properties. In contrast, they seem to be compromised by the occurrence of inflammatory factors inside the degenerated discs. IL-1β was found to slightly promote cell apoptosis and caspase-3 expression, reduce cell proliferation, and reduce the expression of aggrecan and Sox9 in rat NP progenitor cells [50]. Another study indicated that, under inflammatory conditions, NP progenitor cells may undergo neurogenic differentiation; resulting in innervation, a source of discogenic pain [103].

## 13. Discussion

Disc progenitor cells are a newly identified type of progenitor cell, which attract attention regarding the self-repair potential of discs in the case of trauma or ageing; however, their numbers and capacities might be limited due to their harsh microenvironment, and, in consequence, degeneration eventually occurs. Currently, studies on their characteristics and function are sparse. In this study, we revisited the components constituting the disc microenvironment, and analyzed how its changes during degeneration. We reviewed the literature, to see how these factors may affect mesenchymal stem cells, the major type of stem cells currently being intensively investigated for disc repair, combined with the available studies on disc progenitor cells, to shed light on how a degenerated microenvironment may possibly affect disc progenitor cells.

In general, we found that low glucose, acidity, and high osmolarity usually affect cell viability, proliferation, and matrix production. The difference regarding these three factors lies in the effect of low glucose being mild and tolerable, while the effect of acidity and high osmolarity is severe. It is not clear whether acidity and high osmolarity impact cell differentiation potential. On the other hand, matrix stiffness and elasticity mainly regulate the cell differentiation potency, while a softer matrix favors chondrogenesis and stiffer matrices are prone to stimulating osteogenesis. Hypoxia manipulates stem cell properties in multiple ways. It can induce apoptosis, inhibit proliferation, and stimulate chondrogenesis.

From the above analysis, it is clear that the major limiting factors for stem cell survival are high osmolarity and acidity, and the major parameters governing stem cell differentiation are hypoxia and matrix stiffness. Therefore, it is not surprising to see that high osmolarity and low pH surpassed the effects of low glucose in rat BM-MSCs [52].

**Figure 3:**
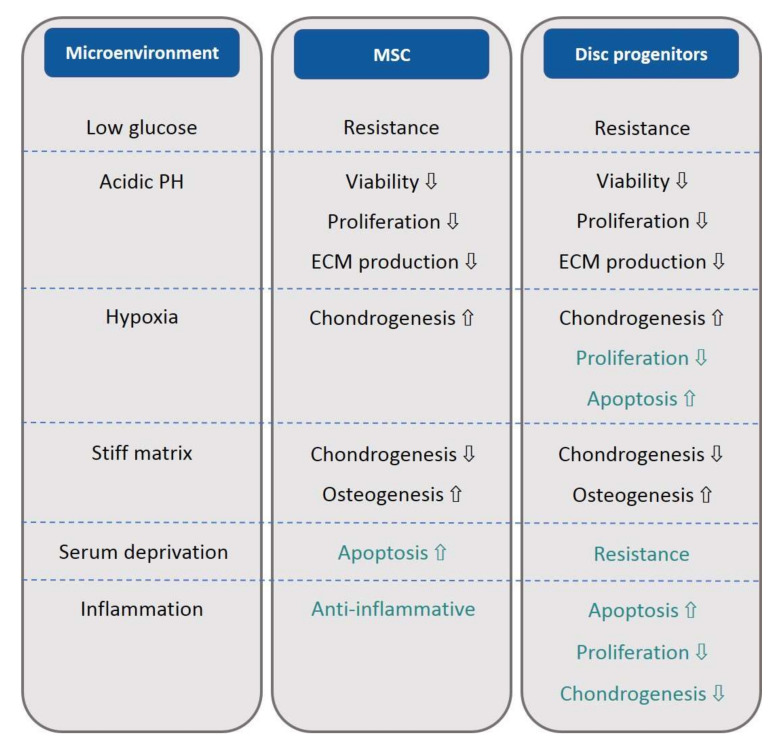
Summary and comparison of the behaviors of MSC and disc progenitors in IVD microenvironment. Behaviors in black indicate similar reactions, while behaviors in green indicate differential reactions, of MSC and disc progenitors to the microenvironmental factors. ECM: extracellular matrix. ⇩ indicates downregulation, while ⇧ indicates upregulation.

When the response of MSCs and disc progenitor cells to factors in the degenerated disc microenvironment were compared, we made some interesting findings (Figure 3). As in MSC-like cells, disc progenitor cells shared several common reactions to the factors constituting the degenerated disc microenvironment with MSCs. They both showed a certain resistance to the limited energy supply caused by low glucose. They both have decreased cell viability and proliferation, and ECM production was inhibited under acidic pH. They both showed enhanced chondrogenesis when subjected to a hypoxic culture. They both displayed inhibited chondrogenic potential and enhanced osteogenic potential in response to a stiffer matrix.

Despite these similarities, MSCs and disc progenitor cells showed several differences in their response to the factors analyzed above. First, disc progenitor cells have a greater resistance to serum deprivation than BM-MSCs, indicating that they have less requirement for the nutrients in serum. Second, MSCs exhibit a certain resistance to the inhibitory proliferation and apoptotic effect of hypoxia, but disc progenitor cells are clearly more vulnerable than MSCs. Third, MSCs are famous for their anti-inflammatory effects and are now widely studied to alleviate tissue inflammation. On the contrary, disc progenitor cells do not seem to possess such a privilege, instead they suffer from inflammation; demonstrating reduced proliferation, more cell apoptosis, as well as reduced chondrogenic marker expression.

An intriguing hint is that a microenvironment close to the native in vivo situation will facilitate the differentiation of MSCs towards the target type of tissue cells. One proof is that mimicking the tendon topography, such as enhancing the substrate modulus, as well as the alignment of type I collagen, promoted the tenogenic differentiation of human MSCs [104]. Some other studies also suggested that adipogenesis and osteogenesis of human MSCs were inclined to occur on substrates with a stiffness close to in vivo microenvironments [105,106]. Considering the native environment that BM-MSCs harbors in bone marrow with sufficient blood supply, whilst NP progenitor cells are in avascular tissue, it is quite understandable that BM-MSCs are more sensitive to serum deprivation than NP progenitor cells; similarly to CEP progenitor cells when compared to NP progenitor cells.

It should be mentioned that not all changes occurring during IVD degeneration are negative for stem cell survival and function. One factor is that hypoxia has a stemness stimulation and chondrogenesis promotion effect on MSCs; furthermore, MSCs are not sensitive to apoptotic induction effects. From this perspective, a deeper hypoxia may be regarded as a positive change. The other factor is that high osmolarity has a negative regulatory role in cell survival and ECM production. Considering that healthy IVD has a high osmolarity, which drops during degeneration, this change could be beneficial to MSCs, and possibly disc progenitor cells.
